# Plasticity of Peripheral Auditory Frequency Sensitivity in Emei Music Frog

**DOI:** 10.1371/journal.pone.0045792

**Published:** 2012-09-18

**Authors:** Dian Zhang, Jianguo Cui, Yezhong Tang

**Affiliations:** 1 School of Automation Engineering, University of Electronic Science and Technology of China, Chengdu, Sichuan, China; 2 Chengdu Institute of Biology, Chinese Academy of Sciences, Chengdu, Sichuan, China; University of Salamanca – Institute for Neuroscience of Castille and Leon and Medical School, Spain

## Abstract

In anurans reproductive behavior is strongly seasonal. During the spring, frogs emerge from hibernation and males vocalize for mating or advertising territories. Female frogs have the ability to evaluate the quality of the males' resources on the basis of these vocalizations. Although studies revealed that central single torus semicircularis neurons in frogs exhibit season plasticity, the plasticity of peripheral auditory sensitivity in frog is unknown. In this study the seasonally plasticity of peripheral auditory sensitivity was test in the Emei music frog *Babina daunchina*, by comparing thresholds and latencies of auditory brainstem responses (ABRs) evoked by tone pips and clicks in the reproductive and non-reproductive seasons. The results show that both ABR thresholds and latency differ significantly between the reproductive and non-reproductive seasons. The thresholds of tone pip evoked ABRs in the non-reproductive season increased significantly about 10 dB than those in the reproductive season for frequencies from 1 KHz to 6 KHz. ABR latencies to waveform valley values for tone pips for the same frequencies using appropriate threshold stimulus levels are longer than those in the reproductive season for frequencies from 1.5 to 6 KHz range, although from 0.2 to 1.5 KHz range it is shorter in the non-reproductive season. These results demonstrated that peripheral auditory frequency sensitivity exhibits seasonal plasticity changes which may be adaptive to seasonal reproductive behavior in frogs.

## Introduction

For seasonal reproductive species, physiology and behavior change substantially across seasons [1], [2]. In songbirds and frogs which use acoustic signals to communicate, the size and morphology of neuronal populations devoted to vocal production change seasonally [3], [4], [5], [6], [7], [8] due to fluctuations in hormone levels including gonadotropins [9], [10]. Studies have revealed dramatic changes in the size of brain areas, number and morphology of neurons across the different seasons [11], [12], [13], [14].

Although many studies have focused on morphological changes in the nervous system associated with seasonal or reproductive state changes in vertebrates, fewer have investigated neurophysiological plasticity. Sisneros and Bass [15] reported that in midshipman fish (*Porichthys notatus*) auditory frequency sensitivity changes seasonally. Lucas et al. [2], [16] found that there are seasonal variations in avian auditory evoked responses to tones. In frogs Goense and Feng [17] showed that single torus semicircularis (TS) neurons display seasonal changes in frequency tuning and temporal properties. However, very little is known about plasticity of auditory brainstem responses (ABRs) in frogs.

In the reproductive season, both male and female frogs must detect and discriminate conspecific vocal signals from heterospecific signals in noisy environments. Moreover, in most frog species the most sensitive frequency responses of the auditory system are tuned to the frequency structure of conspecific calls [18], [19]. In contrast during the non-reproductive season, the frogs become inactive or even hibernate, and there is no adaptive value for extensive auditory processing of complex vocal signals. Thus it is reasonable to hypothesize that the sensitivity of the anuran auditory system should be reduced in the non-reproductive season to save energy.

The Emei music frog, *Babina daunchina*, is a typical seasonal reproductive species that produces complex calls to attract females in the reproductive season and hibernates in the non-reproductive season [20], [21], [22]. The calls of this species have been extensively investigated thus making the species an excellent model for investigating the plasticity of auditory brainstem responses. In the present study, we compared the sensitivity of auditory brainstem responses between the reproductive and non-reproductive seasons in *B. daunchina* to test the hypothesis that the anuran peripheral auditory system exhibits seasonal frequency sensitivity.

## Materials and Methods

### Ethics Statement

This work was conducted with the permission of the Management Office of the Mt. Emei Nature Reserve. All animal procedures were approved by the Animal Care and Use Committee of Chengdu Institute of Biology (permission number: 20110801).

### Animals

During the reproductive season (6^th^ September, 2011), seven male and six female adult *B.daunchina* (body mass 9.02–12.99 g, snout-vent length 4.2–5.9 cm), were captured from ponds on Mt. Emei (29.36 N, 103.22 E) and taken to the Chengdu Institute of Biology for physiological recording. The frogs were maintained in the vivarium with water in a room at 23.5°C with natural photoperiod. The subjects were individually identified by toe-clipping. After recording, the animals were returned to the vivarium and fed crickets until the ABRs were recorded again during the non-reproductive season (February, 2012).

### ABRs measurements

The ABR measurements were conducted in a soundproofed acoustic chamber (5×3.4×2.2 m). The subjects were lightly anesthetized via water immersion (∼3–5 min) with a 0.2% solution of MS-222 (Tricaine Methane Sulfonate). The stimulus presentations, ABR acquisition, equipment control, and data management are similar to that described by Christensen-Dalsgaard [23]. In each subject three 27 gauge stainless steel electrodes (Rochester Electro-Medical, Inc. FL, USA) were inserted subdermally, at the midline above the medulla (about 3 cm caudal to the snout), above the tympanum and in the ipsilateral front leg as inverting, noninverting and ground electrodes, respectively. The recording electrodes were connected to a head stage and amplifier (PA4 & RA4, 20× gain, TDT) via wires wrapped in tin foil.

Stimulus generation and ABR recording were carried out using a digital signal processor RM2 (Tucker-Davis Technologies, Gainesville, USA)), via fiber optic cables linked to RA4 and a USB linked to a laptop computer running custom software (QuickABR) developed by Christian Brandt (University of Southern Denmark, Denmark). Two types of stimuli, tone pips and clicks, were generated by QuickABR and delivered through a portable amplified field speaker (SME-AFS, Saul Mineroff Electronic Inc, USA) which was driven by RM2 and positioned on the table (height: 105 cm) about 110 cm in front of the frog's head. Before ABR recording stimulus levels were calibrated using a G.R.A.S. 46BE 1/4 inch microphone (G.R.A.S. Sound & Vibration, Denmark) with CCP Supply (Type 12AL, G.R.A.S. Sound & Vibration, Denmark) to a 60 decibel sound pressure level (dB SPL re: 20 μPa) positioned at the location of the frog's head. Stimuli were synthesized digitally at octave intervals from 0.2 KHz to 6 KHz, with stimulation duration of 1 ms rise/fall time, 3 ms plateau time and sample rate of 24414 Hz. All biological signals were notch filtered at 50 Hz during data collection.

The ABRs thresholds and latencies were determined using methods similar to that described by Brittan-Powell [24]. Threshold measurements were defined as the lowest stimulus level for which no repeatable responses could be recognized. Threshold measurements were initiated at 90 dB SPL and reduced in 5 dB steps. We assumed the 90 dB level was higher than all ABR thresholds in the Emei music frogs for the stimuli used.

### Analysis and statistics

ABR morphologies, thresholds and latencies obtained from male and female Emei music frogs in response to tone and click stimuli were sorted and analyzed using the SPSS 16.0 statistical program (SPSS Inc., Chicago, IL, USA). Seasonal differences (reproductive season vs. non-reproductive season) were assessed using paired or independent samples *t* tests. In some cases in which data sets failed tests of normal distribution or equal variance and a *t* test could not be used, data were analyzed using the nonparametric Mann-Whitney U test or Wilcoxon Signed Rank Test. For all tests, α was set at 0.05 and data were expressed as Mean ± SD; P<0.05 was considered to be statistically significant.

## Results

### ABRs wave morphology

**Figure 1:**
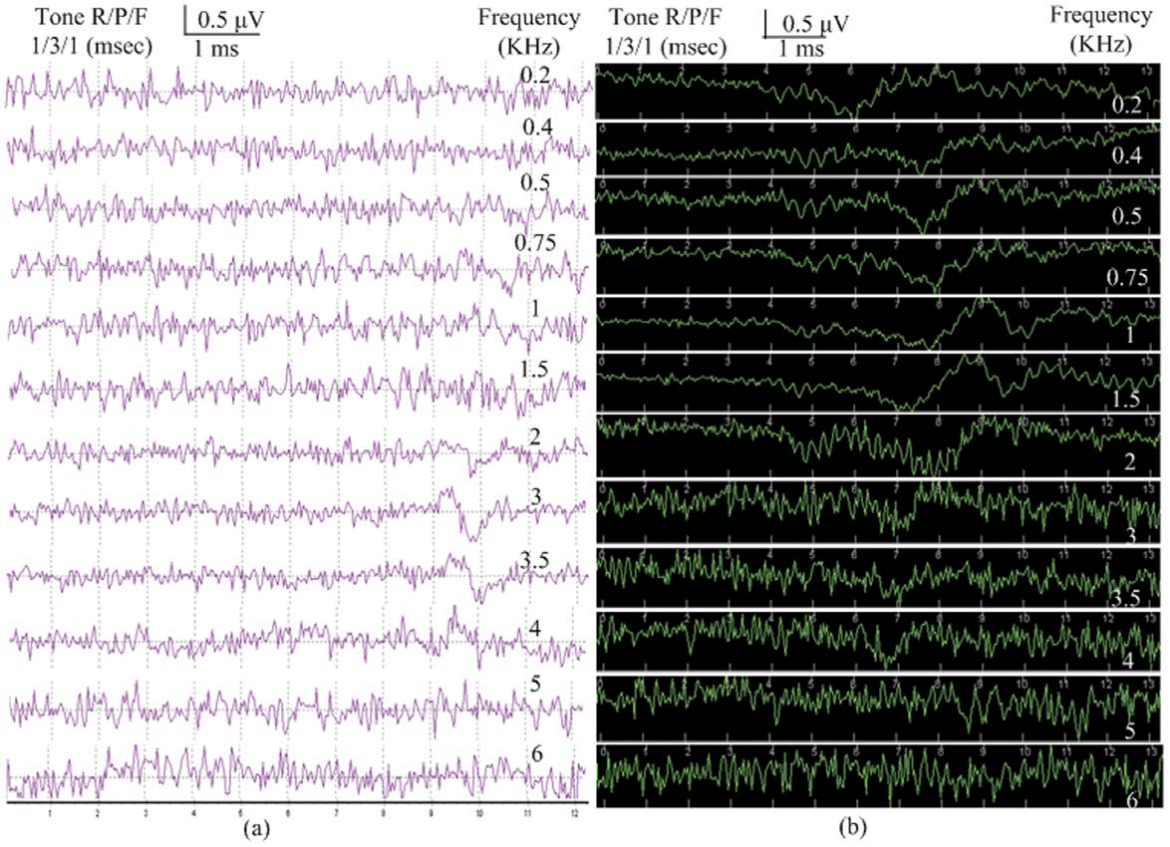
ABR replicates elicited in response to frequency-specific tone pips at 70 dB SPL from the same frog showing valley-peak waveforms in the reproductive season (a) and non-reproductive season (b). Rise, plateau and fall (R/P/F) for each frequency are specified on the left-hand side of the figure.

In both the reproductive and non-reproductive seasons ABRs to tone pip and click stimuli were characterized by valley-peak waveforms, although the waveforms were not obvious at or below 0.5 KHz in the reproductive season. In the non-reproductive season dominant valleys and peaks were easily visualized in all waveforms (Fig. 1). Although ABRs obtained in the reproductive season did not always show distinctive valleys and peaks, ABR wave morphology was identical across the tests in the two seasons.

### ABR thresholds

**Figure 2:**
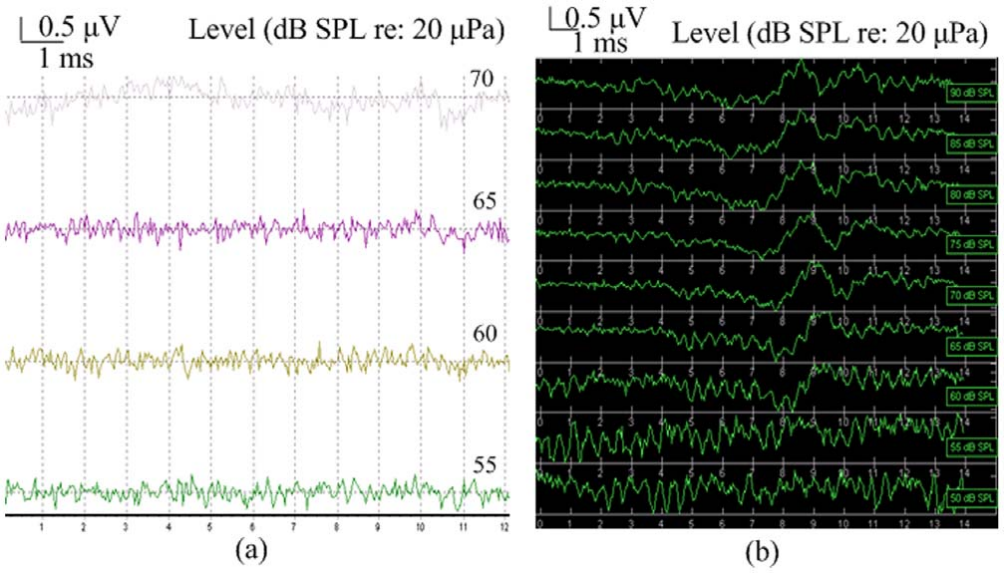
ABRs as a function of stimulus intensity evoked by tone pips of 1 KHz from the same frog in the reproductive season (a) and non-reproductive season (**b**)**.**

**Figure 3:**
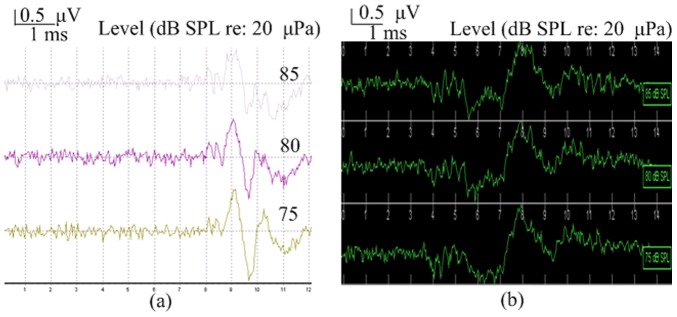
ABRs as a function of intensity evoked by a click stimulus from the same frog in the reproductive season (a) and non-reproductive season (b).

We found that there are apparent threshold differences between the reproductive and non-reproductive seasons across the 0.2 KHz to 6 KHz frequency range. Figure 2 depicts a typical ABR response level series measured from the same male frog evoked by 1 KHz tone pip stimuli for which thresholds of 55 dB SPL and 60 dB SPL were obtained in the reproductive season and non-reproductive season, respectively. As can be seen in Fig. 3, the waveforms evoked by click stimuli tended to have shorter latencies, larger amplitudes and higher thresholds than those evoked by tone pips of 1 KHz or less. As click stimulus intensity increased from 75 to 85 dB SPL, peak amplitudes increased and peak latencies decreased. The possibility that ABR thresholds might differ between males and females was investigated. However, the results are not significantly different between males and females both in the reproductive season and non-reproductive season at the same frequencies (Mann-Whitney U test, P>0.05). So the data from male and female were combined to analyze differences of ABR thresholds between the reproductive season and non-reproductive season.

**Figure 4:**
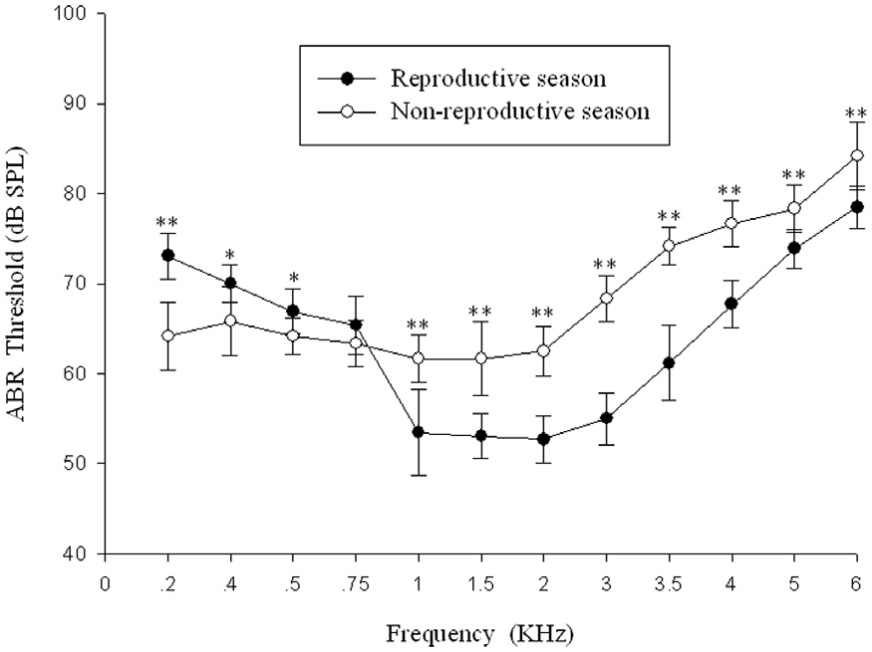
ABR thresholds for Emei music frogs recorded in the reproductive season and non-reproductive season. The points plotted represent the thresholds for tone pips (mean ± SD). *P<0.05, **P<0.01.

ABR thresholds as a function of tone pip frequency are shown in Fig. 4 (Reproductive season: *filled circles*, n = 13; Non-reproductive season: *open circles*, n = 6, the other seven frogs escaped before testing in the non-reproductive season). As can be seen in Fig. 4, in the 1 KHz to 6 KHz range ABR thresholds in the reproductive season are lower than those in the non-reproductive season (Mann-Whitney U test, P<0.01). However, in the 0.2 KHz to 0.5 KHz range ABR thresholds in the reproductive season are higher than those in the non-reproductive season (Mean ± SD, Mann-Whitney U test, P<0.05). Thresholds are lowest in both the reproductive season and non-reproductive season for stimuli in the 1–2 KHz frequency range including stimuli at 1 KHz, 1.5 KHz or 2 KHz stimuli. Similar results were obtained using the Wilcoxon Signed Rank Test (reproductive season, n = 6; non-reproductive season, n = 6, P<0.05).

### ABR latencies

**Figure 5:**
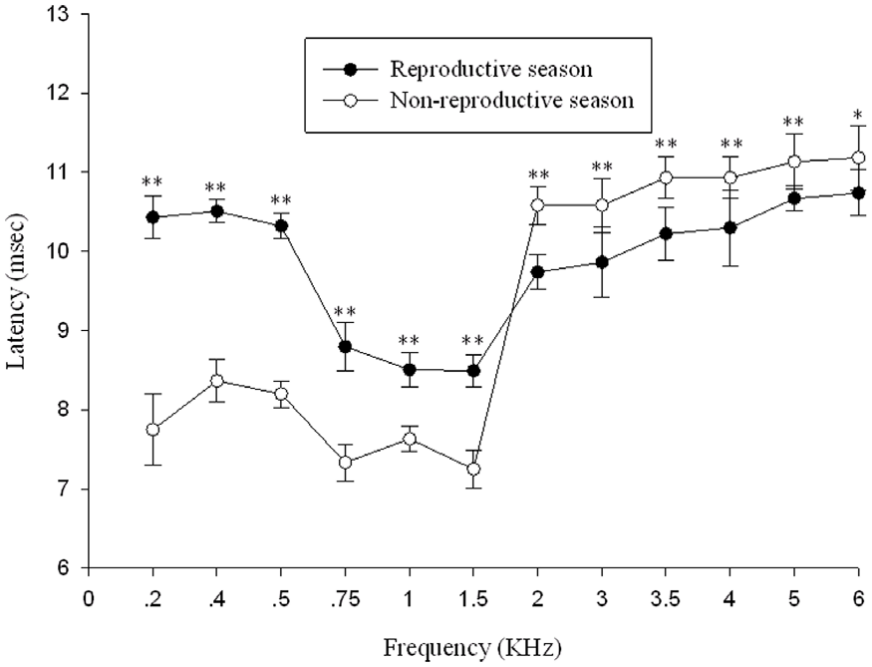
ABR latency to valley as a function of tone pip frequency using threshold stimulation at each frequency. Points represent the latency (mean ± SD). *P<0.05, **P<0.01.

ABR latencies were measured between stimulus onset and the waveform valley (Fig. 1-Fig. 3). There are apparent latency differences between the reproductive and non-reproductive seasons in the 0.2 KHz to 6 KHz range (Fig. 5), both the Wilcoxon Signed Rank Test (6∶6, P<0.01) and Mann-Whitney U test (13∶6, P<0.01) revealed significant differences. As can be seen in Fig. 5 (Reproductive season: *filled circles*, n = 13; Non-reproductive season: *open circles*, n = 6) latencies were longer in the reproductive season than those in the non-reproductive season in the 0.2 to 1.5 KHz range (Mann-Whitney U test, P<0.01). However, ABR latencies are shorter in the reproductive season in the 1.5 to 6 KHz range (Mann-Whitney U test, P<0.01). Latencies were recorded at each frequency using threshold level stimuli which is somewhat different from other methods which have been used [25], [26], although we are aware that latencies typically become shorter as stimulation intensities increase. No significant latency differences were found between males and females both in the reproductive season and non-reproductive season (Mann-Whitney U test, P>0.05).

## Discussion

Although previous studies have shown seasonal plasticity in avian peripheral auditory brainstem responses [2], [16] and in central single torus semicircularis (TS) neurons of frog [17], very little is known about plasticity of peripheral auditory brainstem responses (ABRs) in frogs. The results of the present study show that both ABR thresholds and latencies differ significantly between the reproductive and non-reproductive seasons, and these differences are consistent across the frogs and these patterns could be observed in almost all individuals, indicating that peripheral auditory frequency sensitivity displays seasonal plasticity in Emei music frogs.

The breeding period of *B. daunchina* ranges from May to September depending on the temperature and weather conditions. In the present study, the ABR data for the reproductive season were obtained in September when the field populations were calling or egg laying, while the ABR data for the non-reproductive season were obtained in February when the field populations were hibernating. We found that ABR thresholds are lowest both in the reproductive and non-reproductive season for stimuli in the 1–2 KHz frequency range. These values correspond closely to the range of the dominant frequency band in male advertisement calls [21], [22]. In *Xenopus Laevis* the differences in best hearing sensitivity are in part correlated to variation in middle ear volumes for airborne sounds [25], however, in the Emei music frog, this idea deserves further study.

Both the Mann-Whitney Rank Sum Test (n = 13 for the reproductive season; n = 6 for the non-reproductive season, P<0.01) and the Wilcoxon Signed Rank Test (n = 6 for the reproductive season; n = 6 for the non-reproductive season, P<0.05) showed that the ABR thresholds in the reproductive season are lower than those in the non-reproductive season in the 1 KHz to 6 KHz range. These results strongly support the idea that season changes do affect ABR responses in Emei music frogs, particularly for the frequency band containing the advertisement call dominant frequency. Additionally, there are apparent latency differences between the reproductive season and non-reproductive season in the 0.2 KHz to 6 KHz range (Fig. 5). These results support the idea that the seasonal changes affect ABR latencies in a manner similar to the effect on thresholds, and that the shortest latencies occur in the frequency band containing the advertisement call dominant frequency.

Seasonal plasticity has been reported in the vertebrate nervous system for a number of species. There is evidence in the literature for seasonality in the functioning for both the inner ear [17] and auditory brainstem [2], [14]. The ABR responses in frogs provide an index of auditory function from the level of the cochlea to the auditory brainstem. Our results show that both ABR thresholds and latencies differ significantly between the reproductive and non-reproductive seasons, which are consistent with previous studies of seasonal differences in the auditory midbrain of birds [27], frogs [28], [29] and mammals [26], [30]. During non-reproductive seasons, birds and frogs become inactive, and there is less adaptive value for extensive auditory processing of complex vocal signals. Thus, decreasing auditory sensitivity to save energy may be adaptive in the non-reproductive season. Seasonal changes in nervous system have been associated with hormone levels which are known to fluctuate over the year in seasonal breeders and hibernating animals [9], [10]. However, it is unclear whether auditory frequency sensitivity plasticity in frogs is caused by fluctuations of hormone levels.

In summary, our results show that auditory sensitivity changes seasonally in *B. daunchina* with greater sensitivity for both males and females occurring during the breeding season for frequency bands corresponding to the dominant frequency of male advertisement calls. It is logical to infer that seasonal plasticity in frog hearing represents an adaptation which enhances the detection by both males and females of advertisement or aggressive calls from conspecific males. As in the midshipman fish [15] this phenomenon may reflect the adaptive advantage in *B. daunchina* that reduced auditory sensitivity during the non-reproductive season may save energy when the frogs are relatively inactive or hibernating. Future research is needed to determine the generality of this phenomenon among other vertebrates including mammals.
