# Correction: Plasticity of Peripheral Auditory Frequency Sensitivity in Emei Music Frog

**DOI:** 10.1371/annotation/93c6153d-7ac2-48f3-8aaa-7f464b2d63be

**Published:** 2012-11-19

**Authors:** Dian Zhang, Jianguo Cui, Yezhong Tang

Funding information missing.

The following information was missing from the Funding section: This work was also supported by the National Natural Science Foundation of China (31270042). 

